# Critique of the Chinese dietary guidelines on the consumption of cooking oils

**DOI:** 10.1002/fsn3.1979

**Published:** 2020-11-05

**Authors:** Sisi Yan, Xiangyan Liu, Xin Li, Xiaowen Li, Ji Wang, Lixin Wen

**Affiliations:** ^1^ Laboratory of Animal Clinical Toxicology Department of Clinical Veterinary Medicine College of Veterinary Medicine Hunan Agricultural University Changsha Hunan Province China; ^2^ Laboratory of Molecular Nutrition College of Food Science and Engineering Central South University of Forestry and Technology Changsha Hunan Province China; ^3^ Hunan Collaborative Innovation Center of Animal Production Safety Changsha Hunan Province China

In recent decades, with improved living standards, more attention has been given to dietary intake. Since the first edition of the Chinese dietary guidelines was published in 1989, they have been revised several times based on scientific advances. However, the fourth edition, which was published in 2016, still recommends replacing saturated fatty acids (SFAs) with unsaturated fatty acids (USFAs). The guidelines recommend that SFAs should constitute less than 10% of the total dietary energy intake. Because meat and vegetable oil contain SFAs, the consumption of additional animal fat is not recommended. As a result, the consumption of vegetable oil is on the rise in China, with the use of animal fats in cooking gradually decreasing. However, despite the reduction in the consumption of animal fats, the prevalence of obesity in China has seen an upward trend (Caesar et al., 2016). Based on this phenomenon, we have to point out the irrational recommend that replacing SFAs with USFAs. We expounded existing evidences as below.

It is possible to calculate a rough estimate of the proportion of SFA in the Chinese diet. According to the results of *a survey on the nutritional and health status of Chinese residents* (Chang & Wang, 2016), energy intake from fat was 32.9%, of which 35.9% was derived from animal sources (SFAs proportion approximately 40%–50%) and 64.1% was derived from vegetables (SFAs proportion approximately 15%). Based on these results, the SFAs energy to total dietary energy intake was less than 8%, within the range recommended in the dietary guideline. Dates from another survey showed it was just 7.6%, obviously lower than most of other countries, details show in Table S1. However, decreased consumption of SFAs was not benefit for health seemingly. Compliance with SFAs diet recommendation, prevalence of obesity, and cardiovascular disease increased concurrently (Shen et al., 2017; Zhao et al., 2018).

Weak evidence to support USFAs was superior to SFAs. Historical dietary recommendations were based largely on crude cross‐national studies, short‐term experiments, and animal models. More than this, most animal research was based on an extreme intake model, and how unreasonable this is, nutrient consumption should be above a minimum threshold to avoid deficiency and below a maximum threshold to avoid toxicity. The relationship between nutrient intake and health typically follows a U‐shaped curve. However, media workers usually reported positive effect of USFAs ignored rigorousness of related research. In the past 2 decades, there were numerous of evidence from well‐designed metabolic studies, prospective cohorts, and randomized clinical trials. Several meta‐analysis of prospective cohort studies find no evidence that reduction of SFA may reduce CVD incidence (Harcombe et al., 2017; Siri‐Tarino et al., 2010). Last month, a prospective cohort study involved 2,731 participants showed that higher levels of γ‐linolenic acid, a type of n‐6 polyunsaturated fatty acid (PUFA), were associated with higher type 2 diabetes incidence(Miao et al., 2020).

Considering dietary habits, reduced consumption of lard must accompany with increased consumption of soybean oil in China. Similarly in other countries, consumption of soybean oil less than 0.001 kg/person/year to 12 kg/person/year in the United states during the 20th century (Blasbalg et al., 2011). However, data from animal and clinic experiments indicated that soybean oil maybe has negative effects on our health. It was found that lard promoted fat cell hyperplasia and soybean oil promote fat cell hypertrophy in rodents (Bourgeois et al., 1983). Soybean oil enhanced liver damage induced by dietary cholesterol (Henkel et al., 2018). Soybean oil is more obesogenic than coconut oil and fructose (Deol et al., 2015). Linoleic acid rich in soybean oil was proved inhibited *Lactobacillus reuteri*, and a beneficial bacterium has become less common in Western population (Di Rienzi et al., 2018). A recent randomized controlled trial found evidence that with increased proportion of soybean oil in the Chinese diet, gut microbiota diversity was decreased, and content of indole and arachidonic acid that positively correlated to proinflammatory markers was increased (Wan et al., 2019)..

Health effects of foods rich in SFAs need to be re‐evaluated, included lard, butter, cheese, coconut oil, etc. Several animal studies have shown that SFAs could reduce liver damage, and saturated long chain fatty acids were metabolized by commensal *Lactobacillus* and promote their growth (Chen et al., 2015; Liu et al., 2020). Based on evidence, fatty acid chain length, number of carbon atoms, and the structure of the triglyceride should all be considered and not just the degree of saturation (Dabadie et al., 2005; Decker, 1996; Jenkins et al., 2015; Lieber et al., 1967; Zhang et al., 2019). Lard was a common ingredient in the traditional Chinese diet; however, its use has gradually declined in the past 10 years because of the recommendations contained in the dietary guidelines. The *Compendium of Materia Medica* states that lard can reduce stomach and intestinal bloating and detoxify the liver. Lard has an exceptional triglyceride structure because palmitic acid is esterified in the Sn‐2 position. This specific structure is similar to that of milk fat and plays a key role in lipid digestion and absorption (Decker, 1996; Innis, 2011). Stearic acid in lard mainly esterified in the Sn‐3 position, which was hydrolyzed by pancreatic lipase and lost as a calcium‐fatty acid soap in the feces (Gouk et al., 2014; Lien, 1994). In addition, many other constituents modify the health effects. The medium‐chain and odd‐chain fatty acid content in lard is higher than that in soybean oil and rapeseed oil, and the α‐tocotrienol content in lard is higher than that in other commonly used edible oils (Li et al., 2018). Our studies in rodents (12–16 weeks feeding) provide evidence that lard consumption can help reduce the development of obesity, assuming that fat constitutes 10%–25% of dietary energy intake (Wang et al., 2017). Several systematic reviews of cohort studies suggested a lower risk of stroke with higher consumption of saturated fat (de Souza et al., 2015; Kang et al., 2020).

What we proclaimed is that nutrition of foods needs to be evaluated objectively does not over emphasize they are “good” or “bad,” a moderate amount maybe more appropriate than eating or not eating. In addition, future observational studies should focus on the whole food, instead of single nutrients. When we eat an egg, we do not just take cholesterol after all.

## CONFLICTS OF INTEREST

The authors declare that they have no conflicts of interest.

## AUTHOR CONTRIBUTION

Yan, Sisi, Liu, Xiangyan, and Li, Xin wrote original draft and edited; Li, Xiaowen reviewed manuscript; Wang, Ji provided conceptualization, and Wen, Lixin provided conceptualization and funding acquisition. Wang Ji and Lixin Wen are corresponding author (Wang Ji: wangjics@163.com; Lixin Wen: sfwlx8015@sina.com).

## Supporting information

Table S1
