# Supplementary material for: Critique of the Chinese dietary guidelines on the consumption of cooking oils
Source: Food Sci Nutr. 2020 Nov 5;9(1):583–5. doi: 10.1002/fsn3.1979 (PMC7802536; doi:10.1002/fsn3.1979)
Supplement: Supplementary file 1 — Table S1 [file FSN3-9-583-s001.docx]

Table S1: Data about intake fat/oils in different countries

|  | Total energy (Kcal) | Oils/fats (g) | Energy from fat (%) | Fats from animal (%) | Oils from vegetable (%) | Energy from SFA (%) | references |
| --- | --- | --- | --- | --- | --- | --- | --- |
| China | 2162.3 | 79.7 | 33.2 | 35.9 | 64.1 | <8 | (Chang & Wang, 2016) |
| China | NA | 74.1 | 33.7 | ~20% | ~80% | 7.6 | (Shen et al., 2017) |
| Belgian | 2606.6 | NA | NA | NA | NA | ~14 | (Matthys, De Henauw, Bellemans, De Maeyer, & De Backer, 2006) |
| British | ~2141 | ~78 | 31~35 | NA | NA | 11~13 | (Gibson & Ashwell, 2011) |
| 24 European countries | 1803~2727 | NA | 28.5~46.2 | NA | NA | 8.9~15.5 | (Eilander, Harika, & Zock, 2015) |
| America | 1957.7 | 74.76 | 33 | NA | NA | 11 | (Zhao et al., 2018) |
